# The Impact of Winter Cover Crops on Soil Nematode Communities and Food Web Stability in Corn and Soybean Cultivation

**DOI:** 10.3390/microorganisms12102088

**Published:** 2024-10-18

**Authors:** Jerry Akanwari, Md Rashedul Islam, Tahera Sultana

**Affiliations:** 1 Department of Biological Sciences, Brock University, St. Catharines, ON L2S 3A1, Canada; ja20yb@brocku.ca; 2 London Research and Development Center, Agriculture and Agri-Food Canada, Vineland Station, ON L0R 2E0, Canada; 3 National Microbiology Laboratory, Public Health Agency of Canada, Winnipeg, MB R3E 3R2, Canada; rashedul.islam@phac-aspc.gc.ca

**Keywords:** winter cover crop, soil health, rye, barley, oat, food web, nematode community

## Abstract

There is increasing adoption of winter cover crops (WCCs) in corn and soybean production in Canada, primarily to reduce erosion and increase soil organic matter content. WCCs have the potential to influence nematode communities by increasing free-living nematodes and decreasing plant-parasitic nematodes or vice versa. However, the mechanism by which WCCs change nematode community assemblages still remains a key question in soil food web ecology. We tested the hypothesis that the long-term use of rye (*Secale cereale*), barley (*Hordeum vulgare*) and oat (*Avena sativa*) as monocultures or mixtures promotes nematode communities and improves overall soil health conditions compared to winter fallow. The results from this study revealed that the use of WCCs generally promoted a higher abundance and diversity of nematode communities, whereas plant parasitic nematodes were the most abundant in winter fallow. Moreover, the mixtures of WCCs had more similar nematode communities compared to rye alone and winter fallow. The structure and enrichment indices were higher with WCCs, indicating higher nutrient cycling and soil suppressiveness, which are signs of healthy soil conditions. Furthermore, WCCs significantly reduced the populations of root lesion nematode *Pratylenchus*, although their numbers recovered and increased during the main crop stages. Additionally, mixtures of WCCs promoted the highest abundance of the stunt nematode *Tylenchorhynchus*, whereas winter fallow had a higher abundance of the spiral nematode *Helicotylenchus* during the fallow period and the main crop stages. The results show that the long-term use of cover crops can have a positive impact on nematode communities and the soil food web, but these changes depend on the type of WCCs and how they are used.

## 1. Introduction

Corn (*Zea mays*) and soybean (*Glycine max*) are economically important crops in the Canadian province of Ontario, contributing 64% and 60% to national corn and soybean production, respectively [1]. The quest to increase corn–soybean production to meet the increasing demand requires heavy reliance on pesticides and chemical fertilizers, which have numerous negative environmental impacts [2,3,4,5]. Moreover, this reliance may lead to a decline in microbial biodiversity, increased disease and pest pressure and nutrient runoff [6]. One major concern in Southern Ontario is the substantial evidence linking corn–soybean production to nutrient delivery to the Great Lakes [7,8]. As a result, there has been renewed and increasing interest in promoting crop biodiversity in corn–soybean production.

An alternative to improving crop biodiversity is the use of cover crops due to their numerous benefits [9,10]. It is well documented that cover crops have the potential to influence the soil physicochemical and biological properties [11]. Change in biological communities occurs in response to different qualities and quantities of plant residues as well as root exudates in the soil rhizosphere [12]. Nematodes, along with other microorganisms, are crucial part of soil biological communities whose abundance in soil provides valuable information on the structure, function and resilience of the soil food web [13]. They are abundant across different feeding guilds (herbivores, bacterivores, fungivores, predators and omnivores) [14,15]. Herbivores (plant parasites) are nematodes that feed on plant roots and have been one of the leading causes of crop yield losses [16]. Bacterivores and fungivores feed on bacteria and fungi, respectively, and can contribute to plant-available nitrogen (N) [17]. Predatory nematodes feed on other nematodes, whereas omnivores are generalists with the ability to consume nematodes, fungi and bacteria [15]. The presence of omnivores and predators shows the soil food web is stable and resilient to pest outbreaks [18,19]. Agricultural practices such as use of cover crops or changes in soil environment can affect nematodes numbers across the feeding groups [18]. For example, predators and omnivores are very sensitive to disturbance in the soil whereas bacterivores and fungivores can withstand harsh conditions such as desiccation and anaerobic conditions [14,15]. These characteristics make nematodes useful biological indicators for analyzing the impact of cover crops on soil biodiversity [14].

The most commonly adopted winter cover crops (WCCs) in Ontario are rye (*Secale cereale*), oat (*Avena sativa*) and barley (*Hordeum vulgare*) [4,20]. These cover crops are easy to establish, have superior winter hardiness, abilities to scavenge soil NO_3_ and enhance soil biodiversity [21,22,23]. In Ontario, corn and soybean growers, who are the leading cash crop producers, have increasingly integrated WCCs into their production systems [20]. As Ontario’s corn and soybean growers recognize the benefits of WCCs for soil and environmental protection, it is important to understand the influence of these crops—particularly winter rye—on nematode communities. Research on the impact of WCCs on nematode community composition has been inconsistent. While several studies showed rye as a poor host to some key plant-parasitic nematodes [24,25,26,27], others reported rye to have stimulated a high fungal nematode food web compared to other feeding groups [28]. In contrast, Garba, Stirling [19] showed that winter oat promoted high numbers of bacterivores and omnivores. Studies with winter oat and winter barley have also shown to have variable effects on plant-parasitic nematodes [27,29].

The variability in research findings combined with the increasing adoption of WCCs in corn and soybean production highlight the need to investigate their impact on nematode communities in Ontario. We hypothesize that the use of winter cover crops in corn–soybean production systems provides untapped potential to influence nematode communities and that the effect will vary according to the type of WCC. In the present study, we tested three WCCs used as monocultures or mixtures (rye, oat and barley). Thus, the objectives of this study were to examine: (i) the effectiveness of commonly used WCCs on nematode communities compared to winter fallow, (ii) the long-term effect of WCCs on parasitic nematodes populations, and (iii) the soil health conditions of WCCs compared to winter fallow.

## 2. Materials and Methods

### 2.1. Study Sites

The present study was conducted in Lake Erie region of Southern Ontario, Canada on long term agricultural sites that have integrated WCCs in their soybean–corn production for more than 5 years. The rotational system involved the immediate seeding of WCCs post-harvest of the cash crop (usually in September/October), with termination occurring during the Spring months (April/May). Data were collected from corn–soybean sites that have integrated winter fallow (no WCC), rye, Mixture 1 (rye and barley), and Mixture 2 (oat and rye). Sites were selected to be as similar as possible and were comparable because the main soil management types were fallow and cover crop treatment. Field managements includes fertilization with NPK (15:15:15) and weed and pest control. Cattle manure was occasionally used as soil amendment.

### 2.2. Field Sampling

Soil sampling was carried out in 2021 and 2022 during the cover crop and main crop phases. Soil samples were collected in April at the cover crop phase and in August during the main crop phase of each year. At each study site, four plots (20 m^2^) at a distance of 10 m were selected with similar soil type to minimize the heterogeneity. In each plot, twenty soil cores were collected randomly at 20 cm depth using a standard 2.5 cm diameter soil probe. In cases where there was a high amount of aboveground plant debris, the debris was removed before soil sampling. Collected soil samples from each plot were pooled together and placed in a labelled polyethylene sample bags. All soil samples were stored in a refrigerator at 4 °C until processing.

### 2.3. Nutrient Analysis

During the nematode soil sampling, additional soil samples were collected from each site for nutrient analysis. The samples were sent to A&L Canada Laboratories Inc. for soil nutrient analysis. Detailed nutrients analysis and sites locations are presented in Table S1.

### 2.4. Isolation of Nematodes

Soil samples were gently homogenized and passed through a coarse sieve (mesh size 4 mm) to remove roots, rocks and other coarse materials. Sieves were cleaned between samples by rinsing them under hot tap water. Nematodes were extracted from 100 g subsamples of each soil sample using sugar flotation and centrifugation method [30]. The extracted nematodes were preserved in 5% formalin until morphological analysis.

### 2.5. Morphological Identification of Nematodes

Formalin-preserved nematodes were quantified by counting the number of nematodes under a Leica stereo microscope (MC205 C, Concord, ON, Canada). At least 100 nematodes were randomly picked and identified to the family or genus level using an Omax compound microscope (MD8233S50, United Scope, Irvine, CA, USA) observed at 100 to 400× magnification. The taxonomic identification was performed using the diagnostic keys of Bongers [31] and the University of Nebraska nematode identification website (https://nematode.unl.edu/nemaID.htm, accessed on 2 January 2024). The abundance of each nematode species was adjusted to the number of nematodes per 100 g dry soil.

### 2.6. Nematodes Feeding Group Assignments and Ecological Indices

Nematode taxa were assigned to feeding types—herbivores, fungivores, bacterivores, omnivores and predators—to allow for comparison of their populations. *Tylenchidae* and *Aphelenchoididae* are large feeding groups that may be herbivores, fungivores or both [32,33]. In this study, *Aphelenchoididae* were assigned as fungivores, while *Tylenchidae* were classified as herbivores. Species that could not be assigned were designated as unclassified. All nematode species were assigned to colonizer–persister (cp) values of 1–5 using Nemaplex (http://nemaplex.ucdavis.edu/, accessed on 15 June 2024). Nematodes with a cp value of 1 exhibit high fecundity and are often found in disturbed environments, functioning as enrichment opportunists. In contrast, cp-5 prefers stable environments, has the lowest fecundity, and has longer life cycles [15].

Nematodes ecological indices were calculated using the Nematode Indicator Joint Analysis (NINJA) online tool (https://shiny.wur.nl/ninja/, accessed on 28 June 2024) [34]. The indices include maturity index (MI), which was calculated using free-living nematodes and serves as an indicator of disturbance. Higher values suggest a stable and undisturbed environment, while lower values indicate disturbed environments. Plant-parasitic index (PPI), which is similar to MI but was calculated using herbivores, assumes that their population dynamics reflect the status of plant hosts. Higher PPI values indicate greater plant diversity and a mature herbivore community. Basal index (BI) derives from the relative abundance of stress-tolerant nematode species. Channel index (CI) specifies the degree of fungal feeder participation in the decomposition pathway. Enrichment index (EI) and structure index (SI) reflect food availability and soil food web perturbation, respectively.

### 2.7. Statistical Analysis

All analyses were performed with R software version 4.3.1 [35]. All figures were created using the ggplot2 version 3.5.1 [36]. The effects of WCCs and rotation phase (CC = cover crop/fallow, MC = main crop) on the abundance of nematode communities were estimated using a general linear model (GLM). The varIdent function was used to account for heteroscedasticity within the site and year of rotation. The restricted maximum likelihood (REML) method was utilized for parameter estimation. The forward selection method was used to select the best model with the lowest Akaike information criterion (AIC).

The model was run using the gls function in the ‘nlme’ version 3.1.164 [37]. Analysis of variance (ANOVA) was performed to assess the significance between the treatment groups. Post-hoc pairwise comparisons between treatment (cover crop type) and phase of the rotation were conducted using Tukey contrast with the glht function in the ‘multcomp’ version 1.4.26 [38]. *p*-values < 0.05 were considered significant. Generalized least squares models were also used to analyze the ecological indices of nematodes, as described above. The assumption of homoscedasticity within each treatment was tested by plotting residuals against the means and using Levene’s test for variance in the residuals. The normality of nematode abundance data were checked using a qqnorm plot and using the Shapiro–Wilk test. Data were log (x + 1) and square root transformed for non-normality. We performed Kruskal–Wallis test on data that are not normally distributed using aligned ranks transformation ANOVA (ART-ANOVA) [39].

Non-metric multidimensional scaling (NMDS) ordination based on Bray–Curtis distance was conducted using the ‘vegan’ version 2.6.6.1 to visualize the patterns of nematode communities [40]. Nematode community patterns identified in the NMDS plot were tested for statistical significance using the permutational multivariate analysis of variance (PERMANOVA) of the ‘adonis’ function from the ‘vegan’ package. Variation in nematode communities explained by cover crop treatment and environmental variables was conducted using a partial distance-based redundancy analysis (dbRDA) called by the capscale function in the ‘vegan’ package [40]. The significance of the dbRDA axes was calculated by the axis.long function in the BiodiversityR version 2.16.1 [41]. Nematodes species abundance and environmental variables were standardized using “Hellinger transformation” and the decostand function in the ‘vegan’ package [42]. The significance of the environmental variables and nematodes species were assessed by PERMANOVA at permutations 9999 using the envfit functions in the vegan package [40].

## 3. Results

### 3.1. Effect of Winter Cover Crops on Nematode Community Composition and Diversity

**Figure 1:**
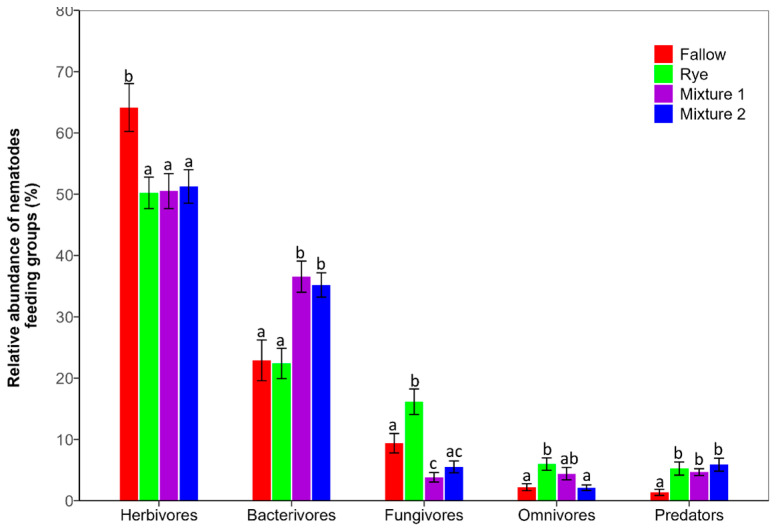
Effect of winter cover crops on nematode community composition in corn–soybean production based on feeding guilds under different treatments. The y-axis represents the relative abundance (%) and the x-axis represents the different feeding groups. The bars are mean ± standard error (n = 4) for each treatment. Different letters indicate significant differences at *p* < 0.05 (Tukey HSD test); Mixture 1 = rye and barley; Mixture 2 = oats and rye.

Sixty-seven nematodes genera belonging to five trophic levels were identified across the study sites (18 herbivores, 17 bacterivores, 10 fungivores, 10 omnivores and 12 predators) (Table 1). *Pratylenchus*, *Heterodera*, *Hoplolaimus*, *Helicotylenchus*, *Mesocriconema*, *Xiphinema*, *Geocenamus*, *Trichodorus* and *Tylenchorhynchus* were the most important plant-parasitic nematodes identified in this study (Table 1). Herbivores (plant-parasitic nematodes) and bacterivores were the most abundant feeding groups (>83%) (Figure 1). The abundance of free-living nematodes was generally higher in the cover crop treatment compared to fallow conditions (Figure 1, Table 1). The use of a mixture of winter WCCs resulted in a statistically significant (*p* < 0.05) increase in bacterivorous nematodes (Figure 1). In contrast, winter rye supported a 46% increase in the populations of fungivores, which was statistically significant compared to both fallow conditions and the mixture of WCCs. The mixture of WCCs exhibited the lowest abundance of fungivores. Additionally, the abundance of predators was highest in the WCC treatments and significantly different from the treatment without cover crops (fallow). The use of WCCs or fallow had impact on the nematodes structural guild, which is ordinate on a 1–5 scale based on “colonizer” (r strategists) to “persister” (K strategists) (Table S2). The cp-2, which is made of bacterivores and fungivores that have relatively high tolerance to adverse conditions compared to cp-1 were the highest in this study (>54%). The cp-1 nematodes (early colonizers) had the second highest relative abundance across all treatment groups.

**Figure 2:**
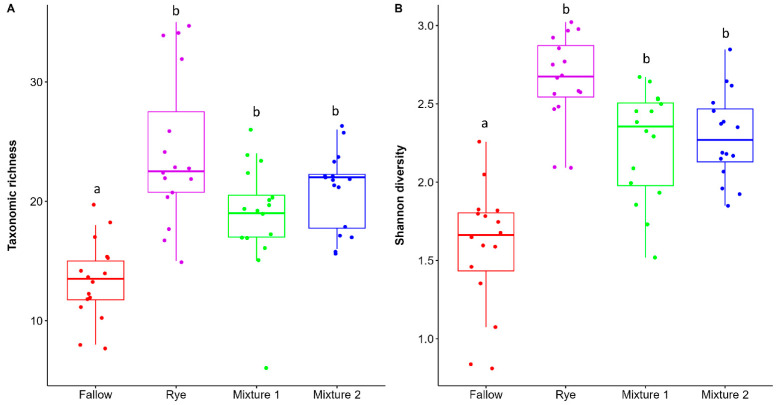
Effect of winter cover crops in corn–soybean rotation on (**A**) nematode taxonomic richness and (**B**) Shannon diversity index. Lower and upper box boundaries are the 25th and 75th percentiles; the line inside the box indicates median; and lower and upper error lines represent 10th and 90th percentiles. Different letters above boxes indicate statistical significance at *p* < 0.05 (Tukey HSD test). Mixture 1 = rye and barley; Mixture 2 = oats and rye.

**Figure 3:**
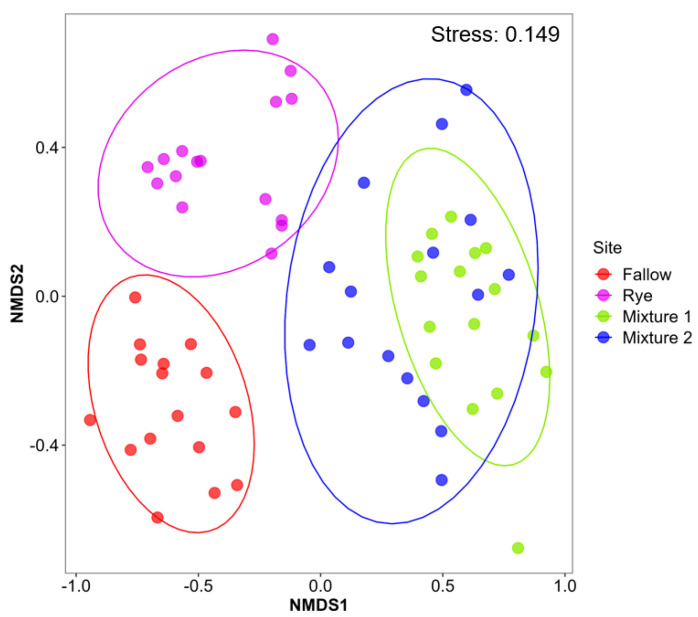
Non−metric multidimensional scaling (NMDS) plot of nematode communities based on Bray–Curtis dissimilarity metric. Circles within the NMDS plot are 90% confidence ellipses. Mixture 1 = rye and barley; Mixture 2 = oats and rye.

**Figure 4:**
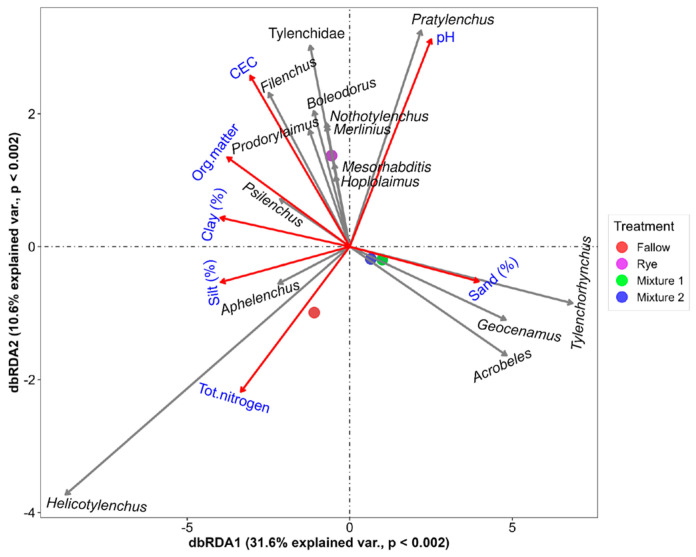
Distance-based redundancy analysis (dbRDA) of the relationship between cover crop treatment, nematode community structure and environmental variables using Bray−Curtis dissimilarity matrix. Only species and environmental factors that explains at least 40% (|r| ≥ 0.40) of the variation and significantly different (*p* < 0.05) are shown. Mixture 1 = rye and barley, Mixture 2 = oats and rye.

The winter fallow significantly decreased the nematodes’ functional richness and Shannon diversity indices (Figure 2). Although, the taxonomic richness and Shannon diversity index did not differ significantly among the cover crops treatments, they were generally higher in the winter rye. The rotation phase did not differ significantly between cover crop and main crop stages (Figure S1). The NMDS analysis using Bray–Curtis distance revealed that WCCs and winter fallow provided habitats to varying abundances of soil nematode community composition (Figure 3). The stress level of 0.149 demonstrated that the use of fallow or cover crop affected the nematodes community structure. The PERMANOVA analysis confirmed that the relative abundance of nematode community composition differed significantly among the treatments (F = 16.755; *p* < 0.0001). Pairwise comparison revealed that WCC nematode communities were significantly different from winter fallow. Mixture 1 and Mixture 2 had the similar community composition (Table S3). The dbRDA supported the differences in soil nematode community structure with the use of winter cover crop and winter fallow in corn–soybean production while also indicating how these changes related to the environmental factors (Figure 4). The envfit function was used to test the significance of each environmental factor (Table S4). The first axis accounted for 31.6% of the variation, and the second axis accounted for 10.6% (Figure 4). *Helicotylenchus* and *Aphelenchus* was associated with total N and silt, which was higher in the conventional fallow. The rye winter cover crop had greater association with environmental variables such as clay, organic matter, cation exchange capacity and pH, and nematode genera such as *Tylenchidae*, *Psilenchus*, *Hoplolaimus*, *Mesorhabditis*, *Merlinius*, *Nothotylenchus*, *Boleodorus*, *Prodorylaimus*, *Filenchus* and *Pratylenchus* (Figure 4). In Mixture 1 and Mixture 2, sand had a strong impact on nematode communities and was positively correlated with *Geocenamus*, *Acrobeles* and *Tylenchorynchus*.

### 3.2. Effect of Winter Cover Crops or Fallow on Plant-Parasitic Nematode and Free-Living Nematode Genera

*Helicotylenchus* (54%), *Heterodera* (4%), *Tylenchorhynchus* (0.33%) and *Pratylenchus* (0.19) were the predominant plant-parasitic nematodes genera in the fallow treatment (Table 1). The relative abundance of *Helicotylenchus* was lower in the first year but increased during the second year (Table S5). The abundance of *Pratylenchus* was significantly lower at fallow and their population also declined over the study period (Table 1 and Table S5). Free-living nematodes (FLN) such as *Cephalobus, Eucephalobus* and *Aphelenchus* were significantly higher in the fallow treatment (Table 1). In the rye treatment, *Helicotylenchus* (11.3%), *Tylenchorhynchus* (1.7%), *Pratylenchus* (11.8%), and *Hoplolaimus* (1.4%) were identified as the most significant plant-parasitic nematodes (Table 1). It was observed that the populations of *Helicotylenchus* were significantly reduced during the first year compared to the second year (Table S5). Also, the mean relative abundance of *Pratylenchus* were lower during the first year than in the second year. The abundance of FLN *Mesorhabditis*, *Ditylenchus*, *Filenchus*, *Nothothylenchus* and *Paraphelenchus* were significantly higher in rye treatment (Table 1). *Tylenchorhynchus*, *Geocenamus*, *Pratylenchus* and *Xiphinema* were significantly higher in Mixture 1 and Mixture 2 compared to fallow (Table 1). Mixture 1 consistently promoted the abundance of *Tylenchorhynchus* over the study the period (Table S4). Furthermore, the relative abundance of *Pratylenchus* was higher during the Mixture 1 phase than at main crop stage (Table S5). Mixture 2 significantly increased the mean relative abundance of *Tylenchorhynchus* by 96% in 2021 and 25% in 2022 (Table S5). Mixture 1 and Mixture 2 significantly increased in the abundance of *Acrobeles* and *Rhabditis* (*p* < 0.05) (Table 1).

### 3.3. Effects of Winter Cover Crops on Nematode Community Indices, Metabolic Footprints and Soil Health Conditions

**Figure 5:**
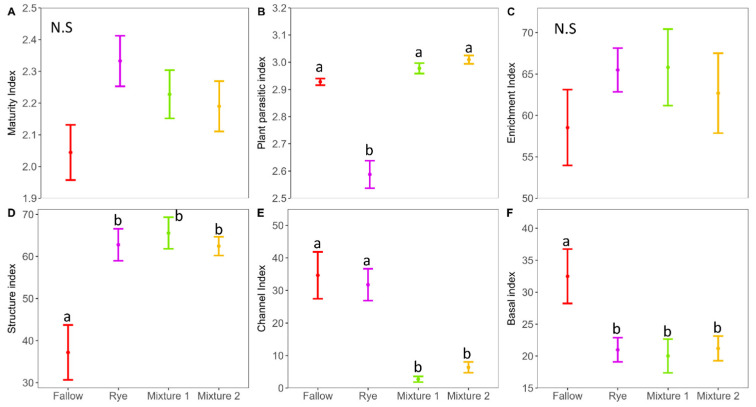
Generalized linear model (GLM) analysis of nematodes community indices ((**A**) = maturity index; (**B**) = plant-parasitic index; (**C**) = enrichment index; (**D**) = structure index; (**E**) = channel index; (**F**) = basal index). The lines are mean and standard errors (n = 4). Different letters represent significant differences among treatments using Tukey HSD at *p* < 0.05. N.S = not significant.

**Figure 6:**
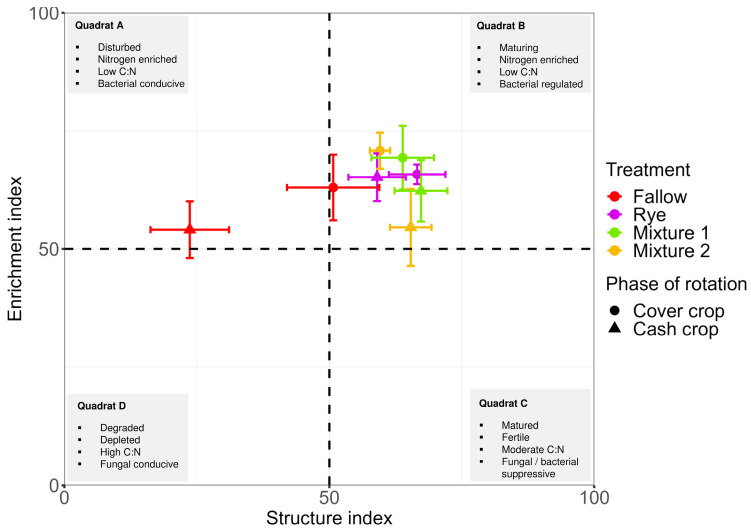
Food web analysis of nematode communities and their positions as soil health indicators. The vertical axis is the enrichment index (nematode reproduction), and the horizontal axis is the structure index (nematode resistance to disturbance). The soil health condition is categorized into four quadrats (A–D). Bars are mean ± standard errors. Mixture 1 = rye and barley; Mixture 2 = oats and rye.

The maturity index (MI) and the structure index (EI) that can be used to measure environmental stability was generally higher with WCCs (Figure 5A, Table S6). In addition, the structure index (SI) was significantly higher with WCCs than winter fallow (Figure 5D, *p* < 0.05). The PPI for winter rye was significantly lower by 22% compared to fallow, Mixture 1 and Mixture 2 (Figure 5B). The CI and BI were generally higher in the winter fallow whereas the WCCs were associated with higher EI (Figure 5C,E,F). A soil nematode faunal analysis using EI and SI, which assess soil health conditions, was shown in Figure 6. Results showed that the soil conditions under WCCs were maturing, an N-enriched, low C/N, bacterial-dominated and regulated system (Quadrant B). Conversely, the winter fallow soil profile revealed a soil health condition that was maturing (Quadrant B) during fallow and disturbed (Quadrant A) during the main crop stage (Figure 6).

**Figure 7:**
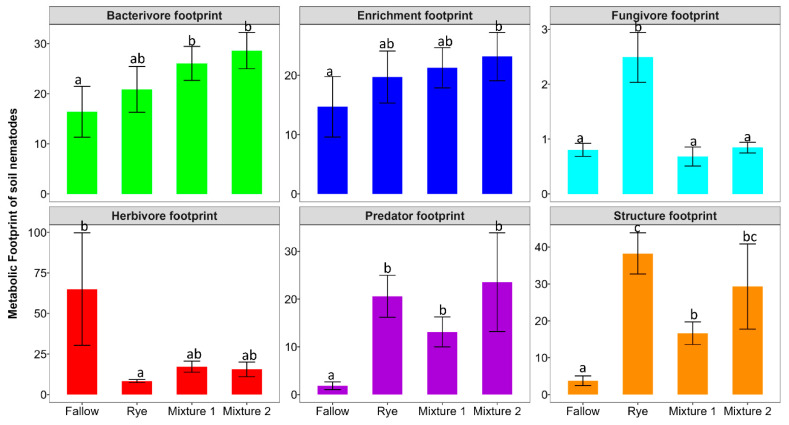
Nematode metabolic footprints under different treatment groups. The bars are mean and standard errors (n = 4). Different letters represent significant differences among treatments using Tukey HSD at *p* < 0.05.

We observed more stability in the soil structure during the winter fallow and rye phase than the main crop phase (Table S6). Conversely, there was higher disturbance during the Mixture 1 and Mixture 2 phases compared to the main crop phase. The metabolic footprints, which is associated with the amounts of carbon and energy entering to the soil food web, was significantly impacted by WCCs (Figure 7). WCCs significantly increased the structure, predator and bacterivore footprint whereas the herbivore metabolic footprint was significantly higher in the winter fallow. Rye treatment was associated with higher fungivore footprints. Although the enrichment footprint was higher in WCCs, only Mixture 2 was significantly higher than in the fallow.

## 4. Discussion

Studies have shown that cover crops can influence the composition, diversity and structure of nematode communities in agro-ecosystems [25,27,29]. In line with the study hypothesis, our results revealed that the cover crops used in this investigation had generally supported a higher abundance and diversity of nematodes compared to the winter fallow. This is because cover crops stimulate microbial growth, and these microbes serve as food for free-living nematodes, leading to an increase in their population [11,43]. This, in turn, tends to have a positive effect on predatory nematodes [44]. However, changes in nematode communities depend on the type of cover crop used and whether it is planted as a single species or in combination with others [19,45,46]. Mixtures of cover crops led to a higher abundance of bacterivores, as reported in previous studies [19,45,47]. According to Melakeberhan and Kakaire [48] and Griffiths [49], bacterivore nematodes can release about 30% of a crop’s nitrogen needs. Therefore, the abundance of bacterivores with mixtures of cover crops suggests faster nutrient cycling and an abundance of mineral nitrogen available for the cash crops [33].

The use of rye as a single cover crop supported the abundance of fungivore nematodes, which is consistent with the study conducted by Gruver et al. [28], which reported higher fungivore populations associated with rye. Omnivores and predators were the most abundant with the use of cover crops, and these nematodes have been used as biological control agents to manage plant-parasitic nematodes [50,51]. A higher abundance of omnivores and predators also indicates that the soil conditions at cover crop sites are conducive for predatory mites and biological control agents, a sign of soil suppressiveness [52]. In the present study, the winter fallow had the lowest abundance of omnivores and predators, which is a concern as this system is more conducive to pathogens and invasive species. In regenerative soil practices, these beneficial nematodes feed on plant-parasitic nematodes and microbivore taxa that graze on bacterivores and fungivores [32].

This study identifies and reports a positive association of free-living nematode genera such as *Rhabditis*, *Eucephalobus*, *Panagrolaimus*, *Plectus*, *Aphelenchoides*, *Cephalobus*, *Aphelenchus* and *Diphtherophora* with WCCs in corn–soybean production. Most of these nematodes have been previously reported as common genera in corn and soybean fields [53,54,55].

Cover crops can reduce plant-parasitic nematode population densities through the release of biofumigants that act as nematocidal compounds [56,57]. It was reported that some cover crops are naturally poor hosts or non-hosts to plant-parasitic nematodes [56,58,59]. Cover crops can also act as trap crops, attracting plant-parasitic nematodes to their roots but preventing the nematodes from completing their life cycle [60]. Moreover, Soriano et al. [61] observed that plant defense hormones from oats could be used to control plant-parasitic nematodes. We did not study the mechanisms of suppression of plant-parasitic nematodes under cover crops. The major plant-parasitic nematode genera detected in this study were *Pratylenchus*, *Heterodera*, *Hoplolaimus*, *Helicotylenchus*, *Mesocriconema*, *Xiphinema*, *Geocenamus*, *Trichodorus* and *Tylenchorhynchus*. Of these seven genera, three (*Pratylenchus*, *Heterodera* and *Xiphinema*) are among the top ten most important plant-parasitic nematodes worldwide [16]. Although Canada has historically had a low incidence of plant-parasitic nematodes [62], recent reports suggest that the incidence is on the rise [63,64].

In this study, *Heterodera*, *Mesocriconema* and *Xiphinema* occurred in low abundance. The PPN genus *Pratylenchus*, the most serious pest in Canada due to its ability to attack most crops, was detected at all study sites [62]. All treatments significantly reduced the populations of *Pratylenchus*, but the numbers were very low in the winter fallow. However, *Pratylenchus* populations increased during the main crop stage, except for the winter fallow, where there was a consistent decrease regardless of the type of crop. This may be due to the cover crops being poor hosts [65], or the nematodes being temporarily immobilized and recovering in the presence of a preferred host (cash crop) [61,66].

Stunt nematodes (*Tylenchorhynchus* and *Geocenamus*) were the most dominant plant-parasitic nematode genera associated with Mixture 1 and Mixture 2. Mixtures of cover crops significantly increased stunt nematode populations, with higher numbers in Mixture 2 during the main crop stage. This suggests that all cover crops were similarly efficient hosts. However, the combination of rye and barley (Mixture 1) lowered the numbers of *Tylenchorhynchus* compared to the main crop stage. *Tylenchorhynchus* is polyphagous and can reproduce on cover crops [24,67]. Contrary to studies suggesting grasses as poor hosts to stunt nematodes (*Tylenchorhynchus* spp.) [65], our study showed that the grasses used are hosts to *Tylenchorhynchus* [67,68] but mixing these cover crops can affect their numbers. As demonstrated by Munawar, Yevtushenko [69], stunt nematodes cause damage to crops similar to other organisms, leading growers to mistakenly attribute symptoms to other pathogens. Further research is needed to determine the impact of stunt nematodes such as *Tylenchorhynchus* on corn and soybean.

In addition, the lack of a suitable host affects nematode populations. Therefore, winter fallow is expected to reduce plant-parasitic nematodes for subsequent crop production [19,70]. In this study, we observed higher plant-parasitic nematode populations associated with winter fallow compared to WCCs. Specifically, we observed higher abundance of *Helicotylenchus* during both the fallow and main crop stages. The possible reason is that crop residues were left on the fallow treatment, providing sufficient host biomass to support plant-parasitic nematodes [70]. This was evident in 2022, where we observed higher abundance of *Helicotylenchus* during fallow, after surviving on corn residues in the winter of 2021.

Furthermore, this study also analyzed the soil food web indices (EI, CI, BI and SI) and metabolic footprints, which are essential for describing soil nutrient mineralization and pest regulation or suppression [71]. The winter fallow site had high CI and BI, indicating fungal-mediated decomposition channels and poor nutrient food web conditions [15,45]. Sites using cover crops generally had high EI and SI, suggesting a system with sufficient resources, an active bacterial decomposition pathway, and greater resilience [15,72]. The soil heath conditions in the cover crop sites during the cover and main crop stages were considered maturing, which is the best conditions for nutrient cycling [73]. The food web condition in the winter fallow sites was characterized by disturbed and maturing conditions. The higher bacterivore and enrichment footprints associated with mixtures of cover crops demonstrate the contributions of nematodes to soil nutrient mineralization. Additionally, the higher structure footprint characterized by the use of WCCs confirms soil suppressiveness. To increase soil suppressiveness in the winter fallow site, a reduction in soil disturbance is required to allow for predatory and omnivore nematodes to thrive [74].

## 5. Conclusions

The present study provides evidence on the effectiveness of cover crops in managing nematode abundances in corn and soybean production in Canada. Winter cover crops increased the abundance and diversity of free-living nematodes, thereby promoting better soil health conditions. Furthermore, this study suggests that incorporating WCCs in corn–soybean cultivation enhances soil nematode metabolic activity by increasing the metabolic carbon of bacterivores and omnivore–predators. As observed in this work, winter fallow was associated with high herbivore pressure, which requires urgent attention to reduce its impact on yield. The use of WCCs, whether as monocultures or mixtures, can have varying impacts on plant-parasitic nematodes. Understanding the host status of cover crops against plant-parasitic nematodes should be considered in the management decision process. Therefore, further investigations are required to fully assess the host status of WCCs used in Canada against individual plant-parasitic nematodes and to evaluate the overall benefits of cover crops on soil health and resilience.

## Figures and Tables

**Table 1:** Relative abundance of soil nematode genera identified in the different treatment (mean ± standard error). Different letters above boxes indicate statistically significant at *p* < 0.05 (Tukey HSD test).

Feeding Guild	Genus	Fallow	Rye	Mixture 1	Mixture 2
Bacterivores	*Acrobeles*	0.79 ± 0.48 ^b^	0.00 ± 0.00 ^b^	9.71 ± 1.18 ^a^	12.05 ± 2.09 ^a^
	*Acrobeloides*	0.00 ± 0.00 ^b^	0.00 ± 0.00 ^b^	0.00 ± 0.00 ^b^	0.26 ± 0.15 ^a^
	*Alaimus*	0.00 ± 0.00 ^b^	0.16 ± 0.08 ^a^	0.05 ± 0.05 ^ab^	0.00 ± 0.00 ^b^
	*Anaplectus*	0.32 ± 0.18 ^b^	0.00 ± 0.00 ^b^	2.23 ± 0.97 ^a^	0.06 ± 0.06 ^b^
	*Cephalobus*	5.35 ± 0.91 ^a^	3.03 ± 0.52 ^b^	1.93 ± 0.50 ^b^	2.65 ± 0.72 ^b^
	*Cervidellus*	0.04 ± 0.04 ^b^	0.07 ± 0.05 ^b^	0.93 ± 0.26 ^a^	0.30 ± 0.12 ^b^
	*Chiloplacus*	1.92 ± 0.57	2.86 ± 0.49	1.85 ± 0.98	2.32 ± 0.65
	*Diplogaster*	0.00 ± 0.00	0.00 ± 0.00	0.39 ± 0.33	0.63 ± 0.40
	*Diplogasteriana*	0.31 ± 0.31	0.00 ± 0.00	0.00 ± 0.00	0.00 ± 0.00
	*Eucephalobus*	2.21 ± 0.68 ^a^	1.07 ± 0.23 ^b^	0.55 ± 0.25 ^b^	0.71 ± 0.23 ^b^
	*Mesorhabditis*	0.00 ± 0.00 ^b^	1.72 ± 0.54 ^a^	0.00 ± 0.00 ^b^	0.04 ± 0.04 ^b^
	*Panagrolaimus*	0.25 ± 0.12	1.18 ± 0.35	1.60 ± 0.79	0.88 ± 0.33
	*Plectus*	2.61 ± 0.61	3.94 ± 0.69	3.81 ± 0.74	2.54 ± 0.52
	*Prismatolaimus*	0.24 ± 0.11 ^a^	0.00 ± 0.00 ^b^	0.00 ± 0.00 ^b^	0.07 ± 0.07 ^ab^
	*Rhabditis*	1.95 ± 0.57 ^b^	1.77 ± 1.01 ^b^	5.58 ± 1.31 ^a^	4.67 ± 1.38 ^a^
	*Tylocephalus*	0.00 ± 0.00 ^b^	0.00 ± 0.00 ^b^	0.61 ± 0.28 ^a^	0.31 ± 0.22 ^ab^
	*Wilsonema*	0.00 ± 0.00 ^b^	0.00 ± 0.00 ^b^	0.06 ± 0.06 ^b^	0.23 ± 0.10 ^ab^
Herbivores	*Aglenchus*	0.00 ± 0.00 ^b^	0.55 ± 0.29 ^a^	0.00 ± 0.00 ^b^	0.00 ± 0.00 ^b^
	*Boleodorus*	0.30 ± 0.26 ^b^	3.67 ± 0.84 ^a^	0.00 ± 0.00 ^b^	0.00 ± 0.00 ^b^
	*Coslenchus*	0.83 ± 0.38 ^b^	3.29 ± 1.55 ^a^	0.06 ± 0.06 ^b^	0.31 ± 0.18 ^b^
	*Geocenamus*	0.00 ± 0.00 ^b^	0.00 ± 0.00 ^b^	13.75 ± 4.07a	13.84 ± 3.86 ^a^
	*Helicotylenchus*	54.38 ± 4.51 ^a^	11.30 ± 2.25 ^b^	0.00 ± 0.00 ^c^	5.20 ± 1.78 ^bc^
	*Heterodera*	4.64 ± 2.23	0.00 ± 0.00	0.98 ± 0.34	0.46 ± 0.32
	*Hoplolaimus*	0.00 ± 0.00	1.42 ± 0.45	0.72 ± 0.47	1.13 ± 0.71
	*Malenchus*	0.00 ± 0.00 ^b^	0.12 ± 0.06 ^a^	0.00 ± 0.00 ^b^	0.04 ± 0.04 ^ab^
	*Merlinius*	0.00 ± 0.00 ^b^	3.67 ± 1.22 ^a^	0.00 ± 0.00 ^b^	0.05 ± 0.05 ^b^
	*Mesocriconema*	0.07 ± 0.07	0.00 ± 0.00	0.17 ± 0.17	0.14 ± 0.08
	*Paratrichodorus*	0.00 ± 0.00 ^b^	0.00 ± 0.00 ^b^	0.00 ± 0.00 ^b^	0.11 ± 0.07 ^a^
	*Paratylenchus*	0.20 ± 0.20 ^b^	0.00 ± 0.00 ^b^	1.80 ± 0.01 ^a^	0.16 ± 0.09 ^b^
	*Pratylenchus*	0.19 ± 0.14 ^b^	11.80 ± 3.24 ^a^	6.69 ± 1.47 ^a^	7.92 ± 1.34 ^a^
	*Psilenchus*	2.24 ± 0.44 ^a^	2.65 ± 0.42 ^a^	0.00 ± 0.00 ^b^	0.28 ± 0.13 ^b^
	*Trichodorus*	0.00 ± 0.00	0.00 ± 0.00	0.39 ± 0.21	0.44 ± 0.18
	*Tylenchorhynchus*	0.33 ± 0.27 ^b^	1.69 ± 0.76 ^b^	25.23 ± 3.83 ^a^	19.70 ± 3.86 ^a^
	*Tylenchus*	0.52 ± 0.21 ^b^	2.38 ± 1.10 ^a^	0.12 ± 0.08 ^b^	0.13 ± 0.09 ^b^
	*Xiphinema*	0.00 ± 0.00 ^b^	0.00 ± 0.00 ^b^	0.36 ± 0.31 ^a^	0.75 ± 0.33 ^a^
Fungivores	*Aphelenchoides*	0.31 ± 0.27	0.78 ± 0.32	0.21 ± 0.14	0.33 ± 0.24
	*Aphelenchus*	6.05 ± 1.31 ^a^	3.02 ± 0.66 ^b^	0.65 ± 0.20 ^bc^	1.57 ± 0.41 ^c^
	*Diphtherophora*	0.00 ± 0.00 ^c^	0.16 ± 0.11 ^c^	1.16 ± 0.32 ^b^	2.08 ± 0.45 ^a^
	*Ditylenchus*	0.00 ± 0.00 ^b^	0.10 ± 0.07 ^a^	0.00 ± 0.00 ^b^	0.00 ± 0.00 ^b^
	*Filenchus*	2.73 ± 0.67 ^b^	7.22 ± 0.64 ^a^	0.17 ± 0.09 ^c^	0.74 ± 0.39 ^c^
	*Leptonchus*	0.00 ± 0.00	0.00 ± 0.00	0.06 ± 0.06	0.04 ± 0.04
	*Nothotylenchus*	0.00 ± 0.00 ^b^	3.92 ± 1.32 ^a^	0.00 ± 0.00 ^b^	0.07 ± 0.07 ^b^
	*Paraphelenchus*	0.00 ± 0.00 ^b^	0.22 ± 0.11 ^a^	0.00 ± 0.00 ^b^	0.11 ± 0.08 ^ab^
	*Tylencholaimellus*	0.06 ± 0.06	0.66 ± 0.32	0.99 ± 0.57	0.26 ± 0.10
	*Tylencholaimus*	0.23 ± 0.16 ^ab^	0.05 ± 0.05 ^b^	0.59 ± 0.27 ^a^	0.11 ± 0.07 ^ab^
Omnivores	*Allodorylaimus*	0.13 ± 0.09	0.16 ± 0.08	0.00 ± 0.00	0.00 ± 0.00
	*Campydora*	0.00 ± 0.00	0.00 ± 0.00	0.00 ± 0.00	0.12 ± 0.12
	*Dorydorella*	0.00 ± 0.00	0.00 ± 0.00	0.05 ± 0.05	0.04 ± 0.04
	*Dorylaimus*	0.04 ± 0.04 ^b^	0.97 ± 0.26 ^a^	0.31 ± 0.31 ^ab^	0.61 ± 0.21 ^ab^
	*Enchodelus*	0.00 ± 0.00 ^b^	0.00 ± 0.00 ^b^	1.29 ± 0.45 ^a^	0.13 ± 0.09 ^b^
	*Epidorylaimus*	0.00 ± 0.00 ^b^	0.19 ± 0.09 ^a^	0.00 ± 0.00 ^b^	0.06 ± 0.06 ^ab^
	*Laimydorus*	0.40 ± 0.23	0.16 ± 0.08	0.34 ± 0.19	0.00 ± 0.00
	*Mesodorylaimus*	0.38 ± 0.16	0.50 ± 0.20	0.06 ± 0.06	0.19 ± 0.15
	*Prodorylaimus*	0.87 ± 0.56 ^b^	3.61 ± 1.14 ^a^	0.06 ± 0.06 ^b^	0.13 ± 0.13 ^b^
	*Pungentus*	0.38 ± 0.16 ^b^	0.37 ± 0.19 ^b^	2.31 ± 0.70 ^a^	0.84 ± 0.33 ^b^
Predators	*Achromadora*	0.00 ± 0.00 ^b^	0.00 ± 0.00 ^b^	0.00 ± 0.00 ^b^	0.09 ± 0.06 ^a^
	*Aporcelaimellus*	0.23 ± 0.13 ^b^	1.74 ± 0.60 ^a^	1.35 ± 0.37 ^ab^	2.14 ± 0.59 ^a^
	*Aporcelaimus*	0.00 ± 0.00	1.05 ± 0.40	1.06 ± 0.34	1.23 ± 0.74
	*Clarkus*	0.41 ± 0.19 ^ab^	0.00 ± 0.00 ^b^	0.78 ± 0.23 ^a^	0.11 ± 0.08 ^b^
	*Discolaimus*	0.00 ± 0.00 ^b^	0.00 ± 0.00 ^b^	0.73 ± 0.29 ^a^	0.37 ± 0.10 ^ab^
	*Eudorylaimus*	0.19 ± 0.15	0.48 ± 0.12	0.29 ± 0.16	0.38 ± 0.19
	*Miconchus*	0.00 ± 0.00	0.00 ± 0.00	0.00 ± 0.00	0.09 ± 0.09
	*Mononchus*	0.13 ± 0.09 ^b^	0.19 ± 0.29 ^a^	0.10 ± 0.10 ^b^	0.09 ± 0.09 ^b^
	*Mylonchulus*	0.08 ± 0.08	0.62 ± 0.29	0.14 ± 0.10	0.47 ± 0.16
	*Nygolaimus*	0.07 ± 0.07 ^b^	0.05 ± 0.05 ^b^	0.00 ± 0.00 ^b^	0.68 ± 0.39 ^a^
	*Prionchulus*	0.25 ± 0.18	0.32 ± 0.09	0.05 ± 0.05	0.09 ± 0.06
	*Pristionchus*	0.00 ± 0.00	0.09 ± 0.05	0.17 ± 0.09	0.13 ± 0.09

Mixture 1= rye and barley; Mixture 2 = oats and rye.

## Data Availability

The raw data supporting the conclusions of this article will be made available by the authors on request.

## Supplementary Materials

The following supporting information can be downloaded at: https://www.mdpi.com/article/10.3390/microorganisms12102088/s1, Figure S1. Comparison of winter cover crop/fallow and main crop phases of corn–soybean production on taxonomic richness and Shannon diversity index. The bars represent mean and standard errors. Bars labeled with the same letter were not significantly different at (*p* < 0.05). Mixture 1 = rye and barley; Mixture 2 = oats and rye. Table S1. Geographical and soil chemical properties of the different sites (treatment). OM = organic matter; CEC = cation exchange capacity; TC = total carbon; TN = total nitrogen; TOC = total organic carbon. Different letters represent significant differences among treatments using Tukey’s honestly significant difference test at *p* < 0.05. Table S2. Percentage of nematodes feeding group and cp values. Table S3. Pairwise nematode compositional dissimilarity between winter fallow and winter cover crops using Bray–Curtis dissimilarity metric. Table S4. Significance of environmental factors based on envfit function statistics in dbRDA analysis. CAP1 and CAP2 are cosine values of the angle between environmental factors and the ranking axis; r2 is the coefficient of determination of environmental factors on species distribution; Pr is the significance test of correlation. Table S5. Variation in the relative mean abundance (%) of some selected nematodes across the cover crop and main crop growing seasons during the two years. WCC = Winter cover crop, MC = Main crop. Table S6. Summary of generalized linear squares model analysis of nematodes community indices and feeding groups among the treatments and phase of the rotation.
