# Supplementary material for: The Impact of Winter Cover Crops on Soil Nematode Communities and Food Web Stability in Corn and Soybean Cultivation
Source: Microorganisms. 2024 Oct 18;12(10):2088. doi: 10.3390/microorganisms12102088 (PMC12045143; doi:10.3390/microorganisms12102088)
Supplement: Supplementary file 1 [file microorganisms-12-02088-s001.zip › microorganisms-3251932-supplementary materials.pdf]

Table S1. Geographical and soil chemical properties of the different sites (treatment). OM = organic matter; CEC = cation exchange capacity; TC = total carbon; TN = total nitrogen; TOC = total organic carbon. Different letters represent significant differences among treatments using Tukey's honestly significant difference test at $p < 0.05$.

Treatment	Latitude	Longitude	Sand (%)	Silt (%)	Clay (%)	pH	OM	CEC (meg/100g)	TC (%)	TN (%)	TOC (%)
Fallow	42.15	−82.81	29.5 ± 0.90 [c]	36.0 ± 0.00 [a]	35 ± 0.77 [a]	5.99 ± 0.19 [c]	3.14 ± 0.06 [b]	11.6 ± 0.54 [b]	1.92 ± 0.17 [ab]	0.78 ± 0.23 [a]	1.87 ± 0.18 [ab]
Rye	43.71	−80.71	38.69 ± 2.43 [a]	29.19 ± 0.64 [b]	32.13 ± 1.79 [b]	7.38 ± 0.04 [a]	3.76 ± 0.04 [a]	20.09 ± 1.56 [a]	2.32 ± 0.08 [a]	0.46 ± 0.13 [ab]	2.19 ± 0.07 [a]
Rey + barley	42.78	−80.61	75.0 ± 0.60 [a]	16.25 ± 0.33 [c]	9.75 ± 0.28 [c]	6.15 ± 0.05 [c]	1.28 ± 0.03 [d]	5.38 ± 0.20 [c]	1.64 ± 0.26 [b]	0.29 ± 0.05 [b]	1.60 ± 0.27 [b]
Rye + oat	42.79	−80.62	77.31 ± 0.95 [b]	14.75 ± 0.60 [d]	7.69 ± 0.42 [c]	6.48 ± 0.10 [b]	1.56 ± 0.06 [c]	6.46 ± 0.54 [c]	1.41 ± 0.17 [b]	0.24 ± 0.04 [b]	1.46 ± 0.15 [b]

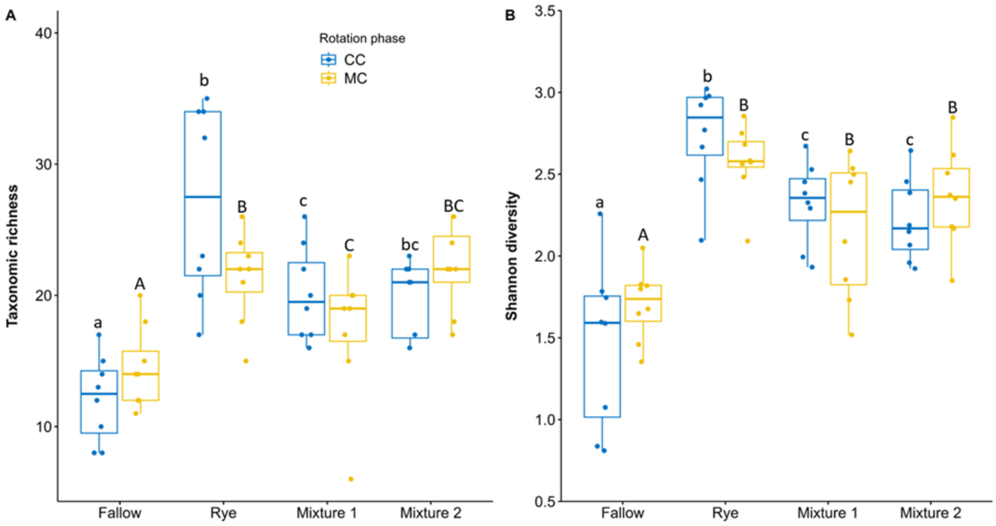

Figure S1. Comparison of winter cover crop/fallow and main crop phases of corn–soybean production on taxonomic richness and Shannon diversity index. The bars represent mean and standard errors. Bars labeled with the same letter were not significantly different at ($p < 0.05$). Mixture 1 = rye and barley; Mixture 2 = oats and rye.

Table S2. Percentage of nematodes feeding group and cp values.

Index Name	Fallow	Mixture 1	Mixture 2	Rye
Free living, % of total	35.9	48.7	49.8	49.5
Herbivores, % of total	64.1	51.3	50.2	50.5
Fungivores, % of total	9.4	5.5	16.1	3.8
Bacterivores, % of total	22.9	35.2	22.4	36.6
Predators, % of total	1.4	5.9	5.3	4.7
Omnivores, % of total	2.2	2.1	6	4.4
CP 1, % of free-living	24.9	27.1	21.1	29.5
CP 2, % of free-living	63.6	51.5	54.4	46.7
CP 3, % of free-living	0.7	4.6	0.3	2.3
CP 4, % of free-living	9.9	8.5	18.5	16.6
CP 5, % of free-living	0.8	8.3	5.7	4.9
PP 2, % of herbivores	6.9	3	39.8	4.4

PP 3, % of herbivores	93.1	94.4	60.2	94.1
PP 4, % of herbivores	0	1.1	0	0.8
PP 5, % of herbivores	0	1.5	0	0.7

Table S3. Pairwise nematode compositional dissimilarity between winter fallow and winter cover crops using Bray–Curtis dissimilarity metric.

Paired Treatment	*F Value*	*p Value*
Fallow vs rye	14.487	0.001
Fallow vs Mixture 1	33.972	0.001
Fallow vs Mixture 2	23.968	0.001
Rye vs Mixture 1	18.959	0.001
Rye vs Mixture 2	12.642	0.001
Mixture 1 and Mixture 2	1.960	0.057

Table S4. Significance of environmental factors based on envfit function statistics in dbRDA analysis. CAP1 and CAP2 are cosine values of the angle between environmental factors and the ranking axis; r2 is the coefficient of determination of environmental factors on species distribution; Pr is the significance test of correlation.

Labels	RDA 1	RDA 2	r2	Pr (>r)
Sand	0.990	−0.1389	0.804	0.0001
Silt	−0.993	−0.115	0.797	0.0001
Clay	−0.993	0.1140	0.791	0.0001
pH	0.664	0.748	0.218	0.0006
Organic matter	−0.939	0.345	0.861	0.0001
Cation exchange capacity (CEC)	−0.755	0.656	0.623	0.0001
Total nitrogen	−0.837	−0.547	0.101	0.0348
Total carbon	−0.871	0.492	0.084	0.076
Total organic carbon	−0.811	0.586	0.062	0.151

Table S5. Variation in the relative mean abundance (%) of some selected nematodes across the cover crop and main crop growing seasons during the two years.

Treatment/Feeding Guild	Genus	WCC 2021	MC 2021	WCC 2022	MC 2022
Fallow					
Bacterivores	*Acrobeles*	0.96 ± 0.96	0.27 ± 0.27	1.74 ± 1.74	0.18 ± 0.18
	Chiloplacus	0.23 ± 0.23 [ab]	2.7 ± 0.59 [ac]	0.00 ± 0.00 [b]	4.75 ± 1.04 [c]
	Eucephalobus	1.31 ± 0.91 [ab]	5.65 ± 1.36 [a]	0.29 ± 0.29 [b]	1.61 ± 0.94 [ab]
	Plectus	3.84 ± 1.31	1.55 ± 1.01	2.89 ± 1.67	2.14 ± 0.92
	Rhabditis	0.45 ± 0.45	3.25 ± 1.47	1.70 ± 1.39	2.41 ± 0.86
Fungivores	*Filenchus*	0.96 ± 0.96	3.23 ± 0.60	1.99 ± 1.99	4.73 ± 1.06
Herbivores	*Helicotylenchus*	42.36 ± 9.49	54.41 ± 8.81	72.81 ± 5.77	47.94 ± 5.51
	Heterodera	7.67 ± 4.43	1.6 ± 1.60	0.25 ± 0.25	9.03 ± 7.60
	Pratylenchus	0.51 ± 51	0.24 ± 0.24	0.01 ± 0.01	0.01 ± 0.01
Rye					
Bacterivores	*Eucephalobus*	1.46 ± 0.65	0.88 ± 0.38	1.07 ± 0.40	0.88 ± 0.51
	Panagrolaimus	1.98 ± 0.72	1.54 ± 0.83	0.22 ± 0.22	1.01 ± 0.75
	Plectus	3.88 ± 0.64	2.86 ± 0.66	5.79 ± 1.56	5.24 ± 1.67
	Rhabditis	2.10 ± 2.10	2.13 ± 2.13	1.31 ± 0.58	5.77 ± 3.56
Fungivores	*Aphelenchoides*	1.64 ± 0.68	0.25 ± 0.25	1.23 ± 0.95	0.00 ± 0.00
	Filenchus	7.63 ± 1.25	8.26 ± 1.90	6.60 ± 1.38	7.39 ± 0.53

Herbivores	*Helicotylenchus*	17.79 ± 0.93 ^{a}	20.71 ± 1.73 ^{a}	6.69 ± 2.00 ^{b}	0.00 ± 0.00 ^{b}
	Pratylenchus	5.97 ± 1.23	6.46 ± 1.91	18.27 ± 10.36	16.52 ± 7.43
Mixture 1					
Bacterivores	*Acrobeles*	7.70 ± 2.46	6.80 ± 2.23	15.80 ± 2.52	12.53 ± 1.54
	Cephalobus	4.02 ± 0.91 ^{a}	1.49 ± 0.40 ^{ab}	2.38 ± 1.11 ^{ab}	0.25 ± 0.25 ^{b}
	Cervidellus	0.24 ± 0.24	1.86 ± 0.83	1.13 ± 0.22	0.50 ± 0.29
	Plectus	6.30 ± 0.53 ^{a}	4.88 ± 1.90 ^{ab}	3.83 ± 0.56 ^{ab}	0.25 ± 0.25 ^{b}
	Rhabditis	18.06 ± 0.74	10.43 ± 1.40	16.07 ± 4.33	11.75 ± 1.44
Fungivores	*Aphelenchus*	1.63 ± 0.43	0.53 ± 0.31	0.32 ± 0.22	0.25 ± 0.25
	Diphtherophora	2.16 ± 0.62	1.04 ± 0.68	1.44 ± 0.58	0.00 ± 0.00
Herbivores	*Heterodera*	0.92 ± 0.67	2.06 ± 1.08	0.70 ± 0.23	0.25 ± 0.25
	Hoplolaimus	0.82 ± 0.49	2.08 ± 1.80	0.00 ± 0.00	3.36 ± 0.54
	Pratylenchus	3.86 ± 1.02 ^{ab}	14.37 ± 2.93 ^{a}	5.81 ± 2.15 ^{ab}	7.01 ± 1.01 ^{b}
	Tylenchorhynchus	8.42 ± 0.32 ^{a}	19.22 ± 1.92 ^{ab}	26.52 ± 3.74 ^{ab}	46.78 ± 3.70 ^{b}
	Xiphinema	0.00 ± 0.00	0.00 ± 0.00	0.20 ± 0.20	1.24 ± 1.24
Mixture 2					
Bacterivores	*Acrobeles*	5.06 ± 1.76	15.7 ± 4.43	12.53 ± 4.38	14.93 ± 4.65
	Cephalobus	1.04 ± 0.41	3.14 ± 1.33	5.21 ± 2.11	1.21 ± 0.41
	Plectus	1.52 ± 0.62	3.39 ± 1.67	3.83 ± 0.58	1.42 ± 0.61
	Rhabditis	5.28 ± 2.33	1.34 ± 1.34	1.82 ± 1.13	10.30 ± 3.54
Fungivores	*Aphelenchus*	6.69 ± 1.38	2.42 ± 0.77	1.37 ± 0.47	0.78 ± 0.45
	Diphtherophora	3.09 ± 0.70	0.54 ± 0.54	2.62 ± 0.89	2.09 ± 1.15
Herbivores	*Helicotylenchus*	1.72 ± 1.72	3.38 ± 2.54	5.27 ± 4.80	10.42 ± 4.12
	Hoplolaimus	0.00 ± 0.00	1.34 ± 1.34	0.35 ± 0.35	2.83 ± 2.59
	Mesocriconema	0.00 ± 0.00	0.17 ± 0.17	0.19 ± 0.19	0.22 ± 0.22
	Pratylenchus	8.69 ± 3.16 ^{ab}	13.73 ± 1.30 ^{a}	2.00 ± 0.82 ^{b}	7.24 ± 0.72 ^{ab}
	Tylenchorhynchus	15.89 ± 3.95 ^{ab}	0.27 ± 0.27 ^{a}	39.06 ± 3.74 ^{b}	23.57 ± 2.69 ^{ab}

Table S6. Summary of generalized linear squares model analysis of nematodes community indices and feeding groups among the treatments and phase of the rotation (CC = cover crop/fallow; MC = main crop).

	**Fallow**	**Rye**	**Mixture 1**	**Mixture 2**	
		Nematode community			
**Maturity Index**					*ANOVA N.S*
WCC	2.12 ± 0.13	2.43 ± 0.09	2.14 ± 0.11	2.04 ± 0.08	*Treatment* ***N.S***
MC	1.97 ± 0.11	2.24 ± 0.13	2.32 ± 0.10	2.34 ± 0.12	*Phase of rotation* ***N.S***
Tukey group ***N.S***					
**Plant-parasitic index**					
WCC	2.94 ± 0.01	2.66 ± 0.04	2.96 ± 0.03	3.02 ± 0.03	*ANOVA* ***
MC	2.92 ± 0.02	2.52 ± 0.09	3.00 ± 0.02	3.00 ± 0.02	*Treatment* ***
Tukey group	a	b	a	a	*Phase of rotation* ***N.S***
**Channel index**					
WCC	31.80 ± 12.20	30.10 ± 4.71	3.90 ± 1.52	4.17 ± 1.48	*ANOVA* ***
MC	37.50 ± 8.47	33.50 ± 8.94	1.50 ± 0.80	8.55 ± 2.88	*Treatment* ***
Tukey group	a	a	b	b	*Phase of rotation* ***N.S***
**Enrichment index**					
WCC	63.00 ± 6.95	65.80 ± 2.07	69.30 ± 6.76	70.80 ± 3.83	*ANOVA N.S*
MC	54.10 ± 6.01	65.20 ± 5.04	62.30 ± 6.51	54.60 ± 8.14	*Treatment* ***N.S***
Tukey group N.S					*Phase of rotation* ***N.S***
**Structure index**					
WCC	50.70 ± 8.68	66.6 ± 5.34	63.80 ± 5.91	59.60 ± 1.91	*ANOVA* ***
MC	23.70 ± 7.42	59.00 ± 5.42	67.30 ± 4.99	65.40 ± 3.92	*Treatment* ***
Tukey group	a	b	b	b	*Phase of rotation N.S*

Values are means (± s.e.m., n = 4) with different letters indicating significant difference ($p < 0.05$:*; $p < 0.01$:**; $p < 0.001$:***). N.S = not significant. Mixture 1 = rye and barley; Mixture 2 = oats and rye. WCC = winter cover crop; MC = main crop
